# Book Review

**Published:** 1917-12

**Authors:** 


**Common Diseases of the Male Urethra.** By FRANK KIDD, M.B., B.C. (Cantab.), F.R.C.S. Pp. vii. 131. London: Longmans, Green & Co. 1917. Price 5s. net.—This will be found a useful book from the general practitioner's point of view. It deals with the scientific treatment of these diseases by methods which can be readily applied; a multitude of remedies, always confusing, are not suggested, but those only which are definitely known to give the best results. For this reason it will be found particularly valuable to those undertaking the treatment of gonorrhœa.

**The Pneumothorax Treatment of Pulmonary Tuberculosis.** By Clive Riviere, M.D. (Lond.), F.R.C.P. Pp. xiii. 180. London: Oxford University Press. 1917. Price 6s. net.—In spite of the frequently supposed view that the production of artificial pneumothorax adds another horror to the history of lung tuberculosis, the author holds that pneumothorax treatment can be regarded as no less than a great and beneficial discovery. It follows that the treatment of pulmonary tubercle by collapse and compression of the diseased lung, through the medium of an artificial or induced pneumothorax, is a procedure which has certainly reached the stage when a convenient text-book should be welcomed. After a history of the method and its technique, the author summarises the general results, and quotes Burnand in saying that "all the physicians who have applied artificial pneumothorax on an adequate scale agree in assigning to it an eminent therapeutic value, and it is capable of bettering very decidedly, perhaps of curing, patients seriously attacked, where no other system of treatment would have a chance of saving them." It is to the *lost* case of phthisis that pneumothorax treatment offers a chance of life and recovered health, and this is its high aim. The book describes fully the indications and contra-indications for this somewhat drastic method.

**The Causation of Sex in Man.** By E. Ramley Dawson. Pp. xii. 226. London: H. K. Lewis & Co. Ltd. 1917. Price 7s. 6d. net.—This second edition describes a new theory of sex based on clinical materials, together with chapters on forecasting or predicting the sex of the unborn child, and in the determination or production of either sex at will. It comes at an opportune time, for owing to the abnormally heavy drain on the manhood not only of this country but of the whole world, the birth of sons becomes a matter of the greatest necessity. The contention of the author is that ovulation occurring *alternately* from the male or right ovary and the female or left ovary, it is possible to correctly forecast the sex of the forthcoming child, and he claims to have had 97 per cent. of successes. The production of either sex at will must consist of avoiding any attempt at fertilisation in the month during which an ovum is produced of the sex which is not desired. We have then to find the ovulation month of the last child, and reckon alternately month by month to find the month which compares in sex to the one which provided the last ovum. The book shows how fallacies may be avoided, but will they?

**Cancer: its Cause and Treatment.** By L. Duncan Bulkley, A.M., M.D. Vol. II. Pp. 282. New York: Paul B. Hoeber. 1917. Price 6s. 6d. net.—Two years ago some new views on this subject were published by the author, and they have received much criticism. He has meanwhile gathered and revealed the unsatisfactory results of the manner of regarding and treating cancer in years past, in the hope and desire that something better may be offered. He claims that cancer should be dealt with from a medical rather than a surgical standpoint, that it is not a purely local disease, that its extension depends largely on the altered condition of modern life along the lines of self-indulgence, that the starting-point of cancer occurs in some cell or cells as a result of local irritation induced by undue ingestion of animal protein, that imperfect intestinal elimination is constantly observed in cancer cases, that it is not contagious or infectious, and that an absolutely vegetarian diet is the first requisition in the treatment and prophylaxis of cancer. Such are a few of the many questions with which the volume deals.

**Acute Appendicitis. Practical Points from a Twenty-five Years' Experience.** By C. Hamilton Whiteford, M.R.C.S., L.R.C.P. Pp. 72. London: Harrison & Sons. 1917. Price 4s. net.—This short account of the diagnosis and treatment of acute appendicitis and its complications abounds in practical instructions communicated in a racy manner. Apt quotations from well-known authorities on appendicitis are freely given, and illustrative cases from the writer's own practice are described to enforce the various principles and methods of treatment advocated. The removal of the inflamed appendix when difficult to detach from an abscess wall is condemned, and the proceeding is also condemned in desperate cases when quick operating for the relief of toxæmia and its attendant collapse is the essential thing. We have read the booklet with pleasure and profit, and can recommend it to all practitioners.

